# Amplified Concern for Social Risk in Adolescence: Development and Validation of a New Measure

**DOI:** 10.3390/brainsci10060397

**Published:** 2020-06-23

**Authors:** Jack L. Andrews, Lucy E. Foulkes, Jessica K. Bone, Sarah-Jayne Blakemore

**Affiliations:** 1 Institute of Cognitive Neuroscience, University College London, London WC1N 3AZ, UK; lucy.foulkes@aeon.co (L.E.F.); sjblakemore@psychol.cam.ac.uk (S.-J.B.); 2 Department of Psychiatry, University College London, London W1T 7BN, UK; jessica.bone.15@ucl.ac.uk; 3 Department of Psychology, University of Cambridge, Cambridge CB2 3EB, UK

**Keywords:** adolescence, social risk, health risk, depression, rejection sensitivity

## Abstract

In adolescence, there is a heightened propensity to take health risks such as smoking, drinking or driving too fast. Another facet of risk taking, social risk, has largely been neglected. A social risk can be defined as any decision or action that could lead to an individual being excluded by their peers, such as appearing different to one’s friends. In the current study, we developed and validated a measure of concern for health and social risk for use in individuals of 11 years and over (N = 1399). Concerns for both health and social risk declined with age, challenging the commonly held stereotype that adolescents are less worried about engaging in risk behaviours, compared with adults. The rate of decline was steeper for social versus health risk behaviours, suggesting that adolescence is a period of heightened concern for social risk. We validated our measure against measures of rejection sensitivity, depression and risk-taking behaviour. Greater concern for social risk was associated with increased sensitivity to rejection and greater depressed mood, and this association was stronger for adolescents compared with adults. We conclude that social risks should be incorporated into future models of risk-taking behaviour, especially when they are pitted against health risks.

## 1. Introduction

Adolescence is a sensitive period of development, characterised by significant changes in both the biological and social environment. In particular, adolescence is a time of social reorientation, greater susceptibility to peer influence and heightened sensitivity to social rejection [1]. Adolescents are also stereotyped as risk takers, which is likely due to evidence that risk behaviours, such as binge drinking, risky driving and smoking, are heightened during this period of life [2,3]. 

This commonly held perspective, that adolescence is a period of heightened risk taking, conceals a more nuanced reality. Social context significantly affects adolescents’ engagement in health risk behaviours. For example, evidence from car accidents shows that, for young drivers, the risk of engaging in a fatal car accident increases with the number of passengers in the car [4]. This is reflected in the experimental literature, with one study finding that, when playing alone, adolescents and adults take a similar number of risks on an incentivised computerised driving task (the stop light task). However, when adolescents played the same driving game in the presence of friends, they took significantly more risks, which was not the case for adults [5]. Adolescents are also more likely to smoke, binge drink and take illicit substances with their peers, compared to when alone [6]. However, not all adolescents take risks, and recent work has led to the suggestion that adolescence is in fact a time of increased sensitivity to risk, characterised by wide variation in risk seeking and risk averse behaviours [7]. Therefore, the extent to which individuals are risk seeking or risk averse depends on multiple factors, including the context of the risk behaviour and the type of risk being taken. 

Sensitivity to the social environment is further reflected in a study that investigated the role of social influence on risk perception in a large sample of individuals ranging from 8 to 59 years. Participants were provided with a set of everyday risky scenarios and were asked to rate the riskiness of each scenario before being shown an average rating purportedly from groups of teenagers or adults, and were then asked to rate again. Whilst all age groups were influenced by the ratings of other people, younger adolescents (12–14 years) were the only age group who were more influenced by the ratings of teenagers than the ratings of adults [8,9]. 

The importance of the peer environment in influencing adolescent decision making is likely tied to evidence showing that during adolescence individuals become increasingly sensitive to social evaluation and social rejection [10,11]. Several studies have shown that adolescents, in comparison to adults, are particularly sensitive to the negative consequences of social rejection. For example, social exclusion can be experimentally-induced by a paradigm called Cyberball, in which participants are either included or excluded by other alleged players in a computerised ball-tossing game. In one study, following the exclusion condition, young adolescents (11–13 years) and mid-adolescents (14–15 years) reported a greater decrease in mood compared with adults (22–47 years; [10]). In another study, adolescents with greater self-reported susceptibility to peer influence took more risks (on a variation of the stop light task, the yellow light game) after being socially excluded (via Cyberball), compared to individuals with high resistance to peer influence [12]. This suggests that individual variation in susceptibility to peer influence can moderate risk taking within a social context amongst adolescents.

Subsequently, it has been proposed that, for some adolescents, the decision to take a health or legal risk (such as smoking) is pitted against a second type of risk: the social risk of being excluded or humiliated [1,13]. A social risk can be defined as any decision or action that could lead an individual to be excluded by their peers, leading to a reduction in one’s social hierarchy or loss of face [13]. This theory proposes that one possible reason that adolescents take health or legal risks is that, in the moment, the social risk is weighted more strongly than potential negative health or legal outcomes [1]. For example, a 14-year-old who is invited to smoke a cigarette by a popular group of friends might weigh the social risk of rejecting the cigarette, and possibly losing face, as greater than the health risks associated with smoking. It is likely, therefore, that individuals with higher sensitivity to social rejection will be more concerned about the social risk involved in any given decision, possibly leading them to take more social risk averse decisions. 

Additionally, high quality friendships and increased social status during adolescence are associated with positive psychological and physical health outcomes [14,15]. Therefore, making decisions that increase one’s social value through reducing exposure to social risk is an important task for adolescents. In addition, there is evidence that feeling dissimilar to one’s peer creates adherence to group norms [16], particularly in individuals who have a significant motivation for group acceptance [17]. It is possible that, for adolescents, this feeling may be particularly marked given the importance that the peer environment has on mental health.

Concerns about social risk may be linked to depressive symptoms. This may be particularly true during adolescence [18], when peer relationships become increasingly important to individuals [19]. According to the social risk hypothesis of depression, some depressed states may emerge as an adaptive, temporary mechanism to minimise the risks associated with social interactions when individuals perceive their value to others to be low and their burden on others to be high [20]. Allen and Badcock define social risks as ‘risks to one’s social circumstances, wellbeing and reputation’ [20]. According to their social risk hypothesis, a loss of significant social relationships and experiencing situations that might lead to reduced social status represent signals that predict social exclusion or ostracism from valuable social contexts [21]. The hypothesis follows that these signals, such as social rejection or loss of social status, orient the individual towards socially risk-averse behaviour. If severe enough for the individual, this may lead to a state of depressed mood. It is likely, therefore, that depression would sit at the extreme end of concern for social risk, when the environmental cues potentially signal that one’s social burden is significantly greater than one’s social value. However, few studies have directly investigated whether adolescence is a period of heightened concern for social risk, and the extent to which concern for social risk predicts depressive symptomatology.

Current questionnaire measures of risk-taking behaviour do not uniformly include social risks as a risk-taking domain, and instead focus on the domains of health (e.g., taking illicit substances), financial (e.g., gambling) or legal (e.g., stealing) risk. One adult risk-taking questionnaire, the Domain-Specific Risk-Taking Questionnaire (DOSPERT) includes a social risk subscale, but this includes items that are not applicable to adolescent populations. For example, the social-risk items in this measure include ‘Approaching your boss to ask for a raise’ and ‘Taking a job that you enjoy over one that is prestigious but less enjoyable’ [22]. Another issue with current questionnaire measures of risk taking is the conflation between health and social risk. Many health risks carry with them some degree of social risk, e.g., smoking may carry with it both health and social risk considerations. Further, it is unclear whether concerns about social risk are independent of concerns for other risk domains, such as health risk behaviours, so whether or not an individual’s propensity to take risks is uniform across risk domains. 

Given these issues, we developed and validated a measure of concern for health and social risk, which is suitable for both adolescents and adults. In this measure, we conceptualised a social risk as any behaviour that marks individuals as being different from their peers—for example, openly endorsing music that friends do not like, or befriending an unpopular peer. We attempted to isolate the social-risk items by including social risks that involve little or no obvious health risk. We conceptualised a health risk as risks to one’s physical wellbeing, such as crossing a street on a red light. We included health risk behaviours that have as little conflation with social risk as possible. 

We had four primary hypotheses. We first hypothesised that concern for social risk would be distinct from health risk concerns. In order to establish this, we developed a measure using exploratory and confirmatory factor analysis (EFA; CFA) to assess whether health and social risk domains are distinct constructs. Second, and in order to validate our measure, we hypothesised that higher concern for social risk would be associated with greater sensitivity to rejection and lower mood. We hypothesised that this relationship would be stronger for adolescents compared with adults. Third, we hypothesised that greater concern for each risk domain would be positively related to risk perception and negatively related to engagement in that risk domain. Finally, we hypothesised that concern for social risk would decrease with age from early adolescence to late adulthood, relative to concern for health risk.

## 2. Method

### 2.1. Participants

***Sample 1 (Exploratory Factor Analysis: EFA; Adults).*** Participants (N = 500) were recruited from two sources: the university participant pool (N = 177) and Prolific, an online participant recruitment and data collection platform (N = 323). Participants (295 females, 204 males, one did not disclose gender) were aged 18–60 years (mean = 32.2, SD = 10.72). 

***Sample 2 (Confirmatory Factor Analysis: CFA; Adults).*** Participants (N = 415) were recruited via Prolific. Participants (284 females, 129 males, two did not disclose gender) were aged 18–77 years (mean = 36.53, SD = 13.10). 

***Sample 3 (Confirmatory Factor Analysis: CFA; Adolescents).*** Participants (N = 484) were recruited from schools in the Greater London area, as part of ongoing research projects in our lab. Participants (333 females, 107 males, four did not disclose gender) were aged 11–17 years (mean = 13.54, SD = 1.91).

All participants were from the United Kingdom and all completed the questionnaires online. Ethical approval was obtained from the university ethics board (7199/001; 3453/001). Participants were paid at a rate of approximately £10 per hour for their time.

### 2.2. Questionnaire Development: The Health and Social Risk Questionnaire (HSRQ) 

We developed a questionnaire measure in order to assess the degree to which adolescents and adults are concerned about engaging in health and social risk behaviours. Given that many social risks also incur health risks, we developed items with as little conflation between the two as possible. We developed a list of social-risk items, e.g., *“spend time with someone your friends don’t like”*, and health risk items, e.g., *“cross a main road when the crossing light is red*”. 

A panel of five researchers with expertise in adolescent social development reviewed an initial list of items and provided feedback on the content and suitability for individuals aged 11 and above, with the aim of making sure each item was distinct from the opposing type of risk. Following this, a total list of 16 items was included in the scale validation: eight health and eight social (see Table 1).

In the version of the questionnaire given to participants, individuals were asked: “For each statement please rate how worried you would feel doing this behaviour. (If you have never done it, imagine how you would feel).” Answers were given on a sliding scale from, “Not worried at all (0)” to “Very worried (100)”. The questionnaire was administered online and the numbers (0–100) were visible along a slider (see supplementary materials for final questionnaire). 

### 2.3. Measures Used for Construct Validation 

All participants completed a number of additional measures in order to assess the construct validity of the HSRQ. All participants included in the adult CFA completed each additional measure (N = 415). However, due to time constraints imposed by testing sessions, a subset of the participants in the adolescent CFA completed the rejection sensitivity (C-RSQ; N = 207) and depressed mood (MFQ; N = 281) measures only.

### 2.4. Rejection Sensitivity 

***Adults: Rejection Sensitivity RS-Adult Questionnaire (A-RSQ).*** The Adult Rejection Sensitivity Questionnaire is a validated measure of sensitivity to actual or perceived rejection [23]. Individuals were presented with nine scenarios such as “You approach a close friend to talk after doing or saying something that seriously upset him/her” and are asked to rate their rejection concern and level of acceptance expectancy. Scores are computed by reversing the level of acceptance expectancy and multiplying this by the level of rejection concern. Scores across the nine items are then averaged to create a total rejection sensitivity score; higher scores indicate higher rejection sensitivity. We hypothesised that higher scores on the social subscale of the HSRQ would be positively associated with higher scores on the A-RSQ. 

***Adolescents: Children’s RejectionSensitivityQuestionnaire (C-RSQ).*** Participants completed the Anxious Expectations subscale of the Children’s Rejection Sensitivity Questionnaire, which is a valid measure of rejection sensitivity in children [24]. Participants were presented with six scenarios and were asked to report on a scale of 0–6 their expected likelihood of the outcome of the scenario and how nervous they would be given the content of the scenario. Their expected likelihood was multiplied by their nervous expectation for each scenario and then a mean score was derived across all items. Higher scores relate to greater rejection sensitivity. We hypothesised that higher scores on the social subscale of the HSRQ would be positively associated with higher scores on the C-RSQ.

### 2.5. Depression

***Adults: Personal Health Questionnaire Depression Scale (PHQ-8).*** The PHQ-8 is a validated eight item measure of depression [25]. Participants were asked how often over the past two weeks they have experienced eight different symptoms, such as “how often were you bothered by feeling down, depressed, or hopeless?” Participants were asked to report on a 4-point scale (0 = “Not at all” […] 3 = “Nearly every day”). We hypothesised that higher scores on the social subscale of the HSRQ would be positively associated with higher scores on the PHQ-8.

***Adolescents: Mood and Feelings Questionnaire (MFQ)—Short Version.*** The MFQ [26] is a depression screening tool for individuals aged 6 to 17 years old. It is a validated measure of depression in children and young people [27]. Individuals were presented with 13 questions, such as “I felt miserable or unhappy” in the past two weeks. Responses were scored on a 3-point scale (0 = “not true”, 1 = “somewhat true”, 2 = “true”). We hypothesised that higher scores on the social subscale of the HSRQ would be positively associated with higher scores on the MFQ.

### 2.6. Social Risk Taking

***Adults: Domain-Specific Risk-Taking (DOSPERT) Scale.*** Participants completed the health and social risk subscales of the 30 item DOSPERT scale, a validated risk-taking measure for adults [22]. Individuals were asked to report on a 5-point scale their likelihood of engaging in each activity or behaviour such as “speaking your mind about an unpopular issue in a meeting at work” (“1= “Very unlikely” to 5= “Very likely”) and their assessment of how risky each situation or behaviour was (“1= “Not at all risky” to 5= “Extremely risky”). We hypothesised that higher scores on the social subscale of the HSRQ would be negatively associated with the social risk engagement subscale of the DOPSERT and positively associated with the social risk perception subscale of the DOSPERT, with the same being true for the health risk subscales. 

***Adolescents.*** Note that adolescents did not complete a social risk-taking measure because the items from the DOSPERT are not appropriate for this age group (e.g., “Approaching your boss to ask for a raise”) and there is no existing social risk-taking measure for adolescents.

### 2.7. Analyses

All data was analysed primarily using the laavan (version 0.6-5), psych (version 1.9.12.3) and semTools (version 0.5-2) packages in R (version 3.62; R Core Team, 2013). 

#### 2.7.1. Exploratory and Confirmatory Factor Analysis

We first conducted an exploratory factor analysis (EFA) using oblique (oblimin) rotation on the initial 16 items relating to health and social risks (eight health, eight social) on a sample of 500 adults. We determined the suitability of our sample size and data for EFA based on the Kaiser–Meyer–Olkin (KMO) index (>0.70) and Bartlett’s test (<0.05) [28]. We determined the number of factors to retain based on examination of the scree plot, retention of factors with eigenvalues of 1 or greater and factors with at least three items. Items with factor loadings of <0.4 were removed. Following factor and item reduction based on the above criteria, we subjected the same data to a confirmatory factor analysis (CFA) to assess the strength of the proposed factor structure. 

We then used CFA to assess the strength of this factor structure in two new samples: one adult group (aged 18–77; N = 415) and one adolescent group (aged 11–17, N = 485). In line with the recommendations outlined by [29], our primary measure of model fit was Root Mean Squared Error of Approximation (RMSEA). An RMSEA of around <0.08 indicates reasonable fit [29]. We also assessed the model fit with the Standardised Root Mean Square Residual (SRMR; <0.08 reasonable fit), Comparative Fit Index (CFI; >0.9 reasonable fit), and the Tucker–Lewis Index (TLI; >0.9 reasonable fit). We computed measures of internal consistency using Cronbach’s alpha and McDonalds omega. We further tested the fit of each two-factor CFA using AIC, by comparing a one-factor solution (where all items are loaded onto one higher order risk factor) with the two-factor solution (health and social risk). A lower AIC represents a better fit to the data. 

#### 2.7.2. Validation and Test–Retest Reliability

To assess convergent and divergent validity, we assessed the relationship between the new HSRQ, rejection sensitivity [23,24] and depressed mood [25,27] across both CFA samples using Pearson r correlations. We then compared the strength of the relationship between the adolescent and adult sample with a Z statistic. One additional risk-taking questionnaire, the DOSPERT [22], was used to relate the HSRQ to risk perception and engagement health and social risks, in the adult sample only. In order to establish the test–retest reliability of the HSRQ, we invited 100 participants from the adult CFA sample to complete the questionnaire a second time 11–12 days after the first completion. We used Pearson r correlations to establish the relationship between these individuals’ scores at time point 1 and 2. 

#### 2.7.3. Age Differences in Concern for Health and Social Risk

Using all the data collected (N = 1399), we computed a mean score of the validated health and social subscale. We determined the relationship between age and the two subscales of the HSRQ using multiple linear regression. We included age, gender and risk domain (health, social) in the model, as well as an age*risk domain interaction, to predict risk concern. We used AIC to compare between linear, quadratic and cubic models, with a lower AIC representing a better fit.

## 3. Results

### 3.1. Sample 1: Exploratory Factor Analysis (EFA)

Analyses showed that the sample size (N = 500) was suitable for conducting factor analysis (KMO = 0.88, Bartlett’s test <0.001). Factor loadings of each item are presented in Table 1. Three factors showed eigenvalues above our threshold of 1: 5.92, 2.53, 1.14, respectively. A fourth factor with an eigenvalue of 0.88 was removed. The third factor (eigenvalue 1.14) only consisted of two items and so was removed. This resulted in a two-factor, 11-item solution. The two factors contained items pertaining to health risks (5 items) and social risks (6 items). We tested the strength of this two-factor solution on the same sample with CFA. The two-factor solution fit the data well (RMSEA = 0.07 (0.06–0.08), SRMR = 0.05, CFI = 0.95, and TLI = 0.93).

### 3.2. Sample 2: Confirmatory factor analysis (CFA; Adult Sample)

We conducted a CFA on a new sample of 415 adults. The sample size was deemed appropriate for testing a model comprising of 24 parameters (11 factor loadings, 11 error variances and 2 factor correlations). The model approximates to a 17:1 subject to parameter ratio, above the recommended 10:1 [30]. The two-factor structure adequately fit the data according to our primary fit index; RMSEA = 0.08 (0.07–0.09). Other model fit indices were good (SRMR = 0.06) or fell just below the suggested cut off (CFI = 0.87 and TLI = 0.83). Factor loadings of each item (see Table 2) were medium to high (0.42–0.76) except for one item (loading of 0.28). Although this item loading was low, we decided to retain it in order to maintain consistency with the factor structure in the adolescent sample and given its good loading in the adult EFA and the adolescent CFA sample. There was a positive correlation between the health and social subscale of the HSRQ (*r*(482) = 0.21, *p* < 0.001). Measures of internal consistency were good (see Table 3).

An additional CFA to assess a one-factor structure did not achieve good model fit (RMSEA = 0.12 (0.11–0.13), SRMR = 0.10, CFI = 0.72, and TLI = 0.70), indicating that concern about risk taking is not a unitary construct and is instead domain specific (health, social). The AIC of the two-factor model (42983.13) was lower than the AIC of the one-factor model (43126.50), suggesting that the two-factor model provided a better fit.

#### 3.2.1. Test–Retest Reliability

To measure the test–retest reliability of the HSRQ, 100 adult participants were invited to complete the questionnaire a second time, 11–12 days later; 68 participants responded. Pearson r correlation between the two time points indicated good test–retest reliability (social risk subscale: *r*(66) = 0.62, *p* < 0.001; health subscale: *r*(66) = 0.74, *p* < 0.001).

#### 3.2.2. Validation

To assess convergent and divergent validity, participants also completed measures of rejection sensitivity (A-RSQ), depressed mood (PHQ-8) and risk taking (DOSPERT).

**Figure 1:**
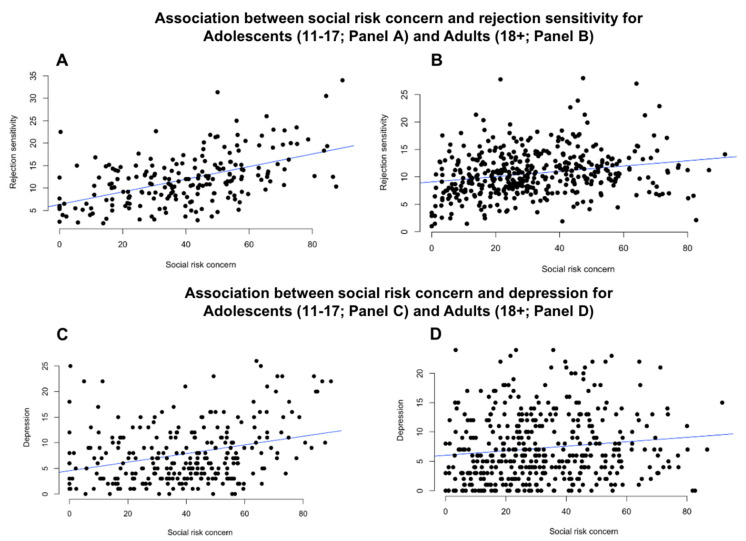
Relationship between concern for social risk and rejection sensitivity for adolescents (*r*(205) = 0.52, *p* < 0.001; panel (**A**) and adults (*r*(413) = 0.22, *p* < 0.001; panel (**B**). Relationship between risk concern and depression for adolescents (*r*(279) = 0.31, *p* < 0.001; panel (**C**) and adults (*r*(413) = 0.13, *p* = 0.009; panel (**D**). The strength of the correlations between concern for social risk and rejection sensitivity and depression was stronger for adolescents than for adults (rejection sensitivity: *Z* = 4.12, *p* < 0.001; depression: *Z* = 2.45, *p* = 0.007).

**Association with rejection sensitivity.** The social risk subscale positively correlated with rejection sensitivity (*r*(413) = 0.22, *p* < 0.001) such that individuals who scored high on concern for social risk also scored high in rejection sensitivity (see Figure 1, panel B). The health risk subscale did not significantly correlate with rejection sensitivity (*r*(413) = −0.00, *p* = 0.99).

**Association with depressed mood.** The social risk subscale positively correlated with depressed mood (*r* (413) = 0.13, *p* = 0.009) such that individuals who scored high on concern for social risk also scored high in depressed mood (see Figure 1, panel D). The health risk subscale did not significantly correlate with depressed mood (*r*(413) = −0.05, *p* = 0.27).

**Association with risk taking.** The social risk subscale of the HSRQ negatively correlated with the likelihood of engaging in social risks subscale of the DOSPERT (*r*(413) = −0.32, *p* < 0.001) and was positively correlated with the perception of social risks subscale of the DOSPERT (*r*(413) = 0.29, *p* < 0.001). In other words, individuals who scored high on concern for social risk on the HSRQ were less likely to engage in social risk behaviours and more likely to rate social risk behaviours as risky. The health risk subscale of the HSRQ was negatively correlated with the likelihood of engaging in health risks subscale of the DOSPERT (*r*(413) = −0.18, *p* < 0.001) and was positively correlated with the perception of health risks subscale of the DOSPERT (*r*(413) = −0.29, *p* < 0.001). Thus, individuals who scored high on concern for health risks were less likely to engage in health risk behaviours and more likely to rate health risk behaviours as risky.

### 3.3. Sample 3: Confirmatory Factor Analysis (CFA; Adolescent Sample)

We conducted a CFA on a new sample of 484 adolescents. The sample size was deemed appropriate for testing a model comprising of 24 parameters (11 factor loadings, 11 error variances and 2 factor correlations). The model approximates to a 20:1 subject to parameter ratio, above the recommended 10:1 [30]. The two-factor structure fit the data well (RMSEA = 0.07 (0.06–0.08), SRMR = 0.05, CFI = 0.95, and TLI = 0.93). Factor loadings of each item were medium to high (0.54–0.79) (see Table 2). There was a positive correlation between the health and social subscale of the HSRQ (*r*(482) = 0.21, *p* < 0.001). Measures of internal consistency were good (see Table 3).

An additional CFA to assess a one-factor structure did not achieve good model fit (RMSEA = 0.18 (0.17–0.19), SRMR = 0.16, CFI = 0.60, and TLI = 0.50), indicating that concern about risk taking is not a unitary construct across domains, and is instead domain specific (health, social), as in the adult sample. The AIC of the two-factor model (49696.51) was lower than the AIC of the one-factor model (50280.89), suggesting that the two-factor model provides a better fit.

Validation

To assess convergent and divergent validity, a subset of the adolescent participants completed measures of rejection sensitivity (C-RSQ; N = 207) and depressed mood (MFQ; N = 281).

***Association with rejection sensitivity.*** The social risk subscale positively correlated with rejection sensitivity (*r*(205) = 0.52, *p*< 0.001) such that individuals who scored high on concern for social risk also scored high in rejection sensitivity (see Figure 1, panel A). The health risk subscale did not significantly correlate with rejection sensitivity (*r*(205) = −0.01, *p* = 0.83).

***Association with depressed mood*.** The social risk subscale positively correlated with depressed mood (*r*(279) = 0.31, *p* < 0.001) such that individuals who scored high on concern for social risk also scored high in depressed mood (see Figure 1, panel C). The health risk subscale did not significantly correlate with depressed mood (*r*(279) = −0.11, *p* = 0.06).

### 3.4. Age Differences in Strength of Correlations

We compared the strength of the correlations between concern for social risk, rejection sensitivity and depression between the adolescent CFA and adult CFA sample. The strength of the correlations between concern for social risk and rejection sensitivity and depression was stronger for adolescents than for adults (rejection sensitivity: *Z* = 4.12, *p* < 0.001; depression: *Z* = 2.45, *p* = 0.007).

### 3.5. Age Differences in Concern for Health and Social Risk

We conducted a multiple regression to assess the relationship between the HSRQ and age, using data collected across all participants (N = 1399; aged 11–77). The outcome was risk concern (i.e., the mean score of the health and social subscales) and the predictor variables were age, gender, risk domain (health, social), and an age by risk domain interaction.

The overall regression model was significant (R^2^ = 0.14, F(3,2793) = 113.2, *p* < 0.001; See Table 4 for estimates). There was a significant main effect of age (β = −0.15; 95% CI: −0.23–0.07; *p* < 0.001) and risk domain (β = −11.69; 95% CI: −15.18–8.19; *p* < 0.001) and a significant interaction between age and risk domain (β = −0.16; 95% CI: −0.27–0.04; *p* < 0.001). There was no main effect of gender (β = 1.07 95% CI: −0.54–2.69; *p* < 0.19).

**Figure 2:**
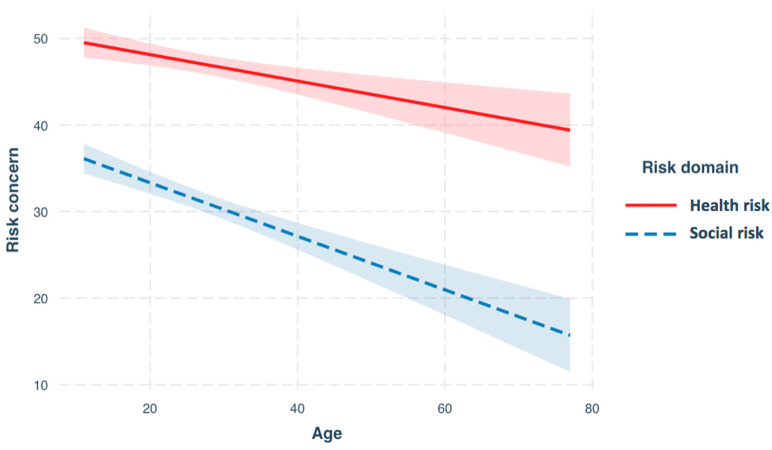
Relationship between age and concern for health risk (slope: β = −0.15, *p* < 0.001) and social risk (slope: β = −0.31, *p* < 0.001). There was a significant difference between the gradient of these slopes (*t*(2794) = 2.7, *p* = 0.008), driven by a steeper decline across age in concern for social risk than for concern for health risk.

To explore the interaction between age and risk domain, we plotted the relationship (Figure 2) and used simple slope analyses. The slope for both risks was significant (social: β = −0.31, *p* < 0.001); health: β = −0.15, *p* < 0.001). There was a significant difference between the gradient of these slopes (*t*(2794) = 2.7, *p* = 0.008), driven by a steeper decline across age in concern for social risk compared to concern for health risk. This linear model (AIC: 25125.34) outperformed a quadratic model (AIC: 25142.67) and cubic model (AIC: 25156.83).

## 4. Discussion

In this study, we developed a questionnaire measure of concern for health and social risk behaviours for use in adolescents and adults. Our results showed that concerns related to engaging in social risks are distinct from concerns related to engaging in health risks. Overall, we found that people reported greater concern for health risk compared with social risk. We investigated age differences in concern for health and social risk, and found that concern for both health and social risk decreased with age, from adolescence through adulthood. However, concern for social risk decreased to a greater extent than concern for health risk. This suggests that, relative to adults, adolescents are more concerned about social risks than health risks.

This heightened concern for social risk in adolescence has implications for understanding why adolescents engage in health and legal risks. One hypothesis is that adolescents are motivated to avoid what they consider to be a greater immediate risk, the social risk of being rejected or excluded by their peers [13]. Avoiding social risks can be considered an important goal during adolescence, a period when social status and friendships provide psychological and physical health benefits [14,15].

The association between our new measure, the Health and Social Risk Questionnaire (HSRQ), rejection sensitivity and depression indicate the potential relevance of social risk for understanding adolescent behaviour and mental health. Individuals who report greater concern for social risk were more likely to report greater sensitivity to rejection (Adolescents: C-RSQ; Adults: A-RSQ). Social rejection is an unpleasant feeling and therefore it makes sense that individuals with a heightened degree of sensitivity to the negative effects of social rejection would be more concerned with engaging in situations that could lead to, or indicate a possibility of, social rejection. Within the adult sample, individuals who scored high on concern for social risk were less likely to engage in socially risky behaviours and were more likely to rate social risk behaviours as risky. This finding indicates that higher concern for social risk is related to an increase in rejection sensitivity and an increase in socially risk-averse behaviour.

Concern for social risk was also related to depressive symptomatology (Adolescents: MFQ; Adults: PHQ-8), such that individuals with greater concern for social risk were more likely to report higher levels of depressive symptoms. This finding supports the predictions made by the social risk hypothesis of depression [20]. This hypothesis proposes that, when cues in the environment signal that one’s social burden is significantly greater than their social value, depression manifests as an adaptive mechanism to remove the individual from social situations which might confer further risk of social rejection.

We showed that concern for social risk was more strongly associated with rejection sensitivity in adolescents (11–17 years), than in adults (18+ years). During adolescence, individuals are particularly sensitive to social evaluative concerns [11], and peer perceptions influence adolescents’ social and personal worth [31]. Adolescents are also hypersensitivity to social rejection relative to adults [10]. This fits with our finding that concerns for social risk are more tightly linked to rejection sensitivity among adolescents, relative to adults. In addition, and as previously discussed, adolescents with good quality friendships and higher social status have more favourable psychological and physical outcomes later in life. Thus, it is potentially beneficial and adaptive for adolescents to try to avoid the risk of social rejection [14,15].

Additionally, the association between concern for social risk and depressive symptoms was stronger in adolescents than adults. This suggests that the social environment may be particularly salient for mental health during this developmental period [18,32]. This is important because the incidence of many mental health problems, including depression, increases significantly during adolescence [33].

Our findings have a number of implications. At the theoretical level, the way in which risk behaviours have been traditionally conceptualised has focused heavily on the health, financial, legal and recreational domains. Our results suggest that social risk should be incorporated into our understanding of risk-taking behaviour. For some individuals, taking a social risk, and placing themselves at risk of social rejection, is a real and ‘risky’ decision. At the practical level, interventions aimed at reducing health and legal risk behaviours should recognise the importance of concerns surrounding social risks. One promising approach is to focus on peer-led interventions, which work to influence social norms surrounding unhealthy or illegal behaviours [34]. This approach encourages healthy behaviours by reducing the social risk of being ostracised by peers. Interventions using a peer-led approach have shown positive results for unhealthy behaviours such as bullying [35] and smoking [36].

## 5. Limitations

The HSRQ is a valid measure for individuals aged 11+. However, this measure has not been validated for children below 11 and very little is known about social risk in this younger age group. Future work should explore the extent to which the current items and factor structure are valid for use in children below this age. Additionally, we did not test the relationship between our measure of concern for social risk and engagement in real-life social risks in the adolescent sample (11–17 years) because of a lack of appropriately validated scales for this age group. This is a limitation when making comparisons with the adult sample (18+) and future work should explore the relationship between our concern for social risk measure and engagement in real-life social risks among adolescents.

Further, our sample was collected from the United Kingdom and therefore this measure should be cross-culturally validated for use in other socio-cultural environments. In addition, the HSRQ is based on self-report, and an important line of subsequent work is to relate responses on this questionnaire measure to a task-based assessment of social risk. Finally, the present study was not designed to investigate the degree to which individuals weigh up the health vs. social consequences of a given ‘risky’ decision. Therefore, an important outstanding question is the degree to which individual variation in concern for health and social risk impacts involvement in ‘risky’ behaviours, especially when individuals are presented with risks that often carry both social and health consequences, such as smoking or dangerous driving.

## 6. Conclusions

In the current study, we developed a self-report measure of concern for health and social risk for use with adolescents and adults. We found that heightened concern for social risk was related to increased sensitivity to rejection and depression, with this relationship being stronger for adolescents compared to adults. This supports the body of evidence that adolescence is a period of heightened sensitivity to the social environment. In addition, both concern for health and social risk decreased with age, but the rate of decrease was steeper for social versus health risk, suggesting that adolescence is a period of amplified concern for social risk. Practically, these findings have potential implications for policy. Within an educational context, an understanding of social risk may offer insight into why adolescents are more or less motivated to engage with school work. For example, if individuals who try hard at school are perceived as unpopular or uncool, then being openly motivated in the classroom could be a social risk [37]. Within a legal context, concerns surrounding social risk may be a factor in adolescents’ decisions to engage in criminal behaviour, particularly in peer contexts when opting out of a group behaviour could risk being excluded from the group. Together, these findings highlight the importance of social risk in adolescent behaviour and suggest that interventions to reduce risk-taking behaviours in this age group should consider the role of social risk.

## Figures and Tables

**Table 1:** Item loadings from the exploratory factor analysis (EFA).

Risk Item	Factor 1	Factor 2	Factor 3	Factor 4
1. Defend an unpopular opinion that you believe in.	0.86			
2. Admit that you listen to a singer or band that none of your friends like.	0.65			
3. Argue with a popular friend in front of a group of people.	0.83			
4. Wear clothes that are really different to your friends’ clothes.	0.5			
7. Stand up for someone who is being mocked by your friends.	0.76			
8. Spend time with someone your friends don’t like.	0.56			
9. Eat food that has passed its sell-by date.		0.57		
10. Ride a bicycle without wearing a helmet.		0.48		
13. Cross a main road when the crossing light is red.		0.64		
15. Pick up broken glass with bare hands.		0.78		
16. Drink tap water in a foreign country.		0.56		
5. Miss a popular friend’s party that lots of people are attending.			0.93	
6. Choose to stay at home when your friends are going out.			0.8	
11. Spend an afternoon in the sun without wearing sun cream.				0.35
12. Eat unhealthy (high fat/sugar content) foods.				0.66
14. Avoid doing regular exercise.				0.78

**Table 2:** Item loadings from the confirmatory factor analysis (CFA) in both the adult and adolescent sample.

Risk Item	Adult (CFA)		Adolescent (CFA)	
	Social	Health	Social	Health
1. Defend an unpopular opinion that you believe in.	0.6		0.77	
2. Admit that you listen to a singer or band that none of your friends like.	0.7		0.67	
3. Argue with a popular friend in front of a group of people.	0.44		0.79	
4. Wear clothes that are really different to your friends’ clothes.	0.63		0.59	
7. Stand up for someone who is being mocked by your friends.	0.76		0.69	
8. Spend time with someone your friends don’t like.	0.64		0.58	
9. Eat food that has passed its sell-by date.		0.42		0.54
10. Ride a bicycle without wearing a helmet.		0.6		0.7
13. Cross a main road when the crossing light is red.		0.62		0.77
15. Pick up broken glass with bare hands.		0.53		0.73
16. Drink tap water in a foreign country.		0.28		0.54

**Table 3:** Measures of internal consistency.

	Social		Health	
	Cronbach’s Alpha	McDonalds ω	Cronbach’s Alpha	McDonalds *ω*
CFA (Adults)	0.79	0.8	0.62	0.63
CFA (Adolescents)	0.84	0.84	0.79	0.79

**Table 4:** Estimates from the model predicting risk concern.

Predictor	*β*	SE	*t*	*p*
Intercept	49.43	1.85	26.69	<0.001
Age	−0.15	0.04	−3.68	<0.001
Risk domain (social risk)	−11.69	1.78	−6.55	<0.001
Gender	1.07	0.82	1.31	0.19
Age * risk domain (social risk)	−0.016	0.06	−2.7	0.008

**Note:** * = an interaction term; *β =* beta coefficient; SE = standard error; *t* = t statistic (the *β* divided by the SE); *p* = significance.

## Supplementary Materials

The following are available online at https://www.mdpi.com/2076-3425/10/6/397/s1, Table S1: Health and Social Risk Questionnaire (HSRQ).
