# Supplementary material for: Amplified Concern for Social Risk in Adolescence: Development and Validation of a New Measure
Source: Brain Sci. 2020 Jun 23;10(6):397. doi: 10.3390/brainsci10060397 (PMC7349691; doi:10.3390/brainsci10060397)
Supplement: Supplementary file 1 [file brainsci-10-00397-s001.pdf]

Supplementary material

Health and Social Risk Questionnaire (HSRQ)

For each statement please rate how worried you would feel doing this behaviour? (If you have never done it, imagine how you would feel). Not worried at all (0) – Very worried (100)*

1. Defend an unpopular opinion that you believe in. (S)
2. Admit that you listen to a singer or band that none of your friends like. (S)
3. Argue with a popular friend in front of a group of people. (S)
4. Wear clothes that are really different to your friends' clothes. (S)
5. Stand up for someone who is being mocked by your friends. (S)
6. Spend time with someone your friends don't like. (S)
7. Eat food that has passed its sell-by date. (H)
8. Ride a bicycle without wearing a helmet. (H)
9. Cross a main road when the crossing light is red. (H)
10. Pick up broken glass with bare hands. (H)
11. Drink tap water in a foreign country. (H)

Subscales: (S) = Social, (H) = Health.
Scoring: Compute an average score for each subscale by summing the responses of the social items and dividing them by six, and summing the responses of the health items and dividing them by five.

*Present as a sliding scale
